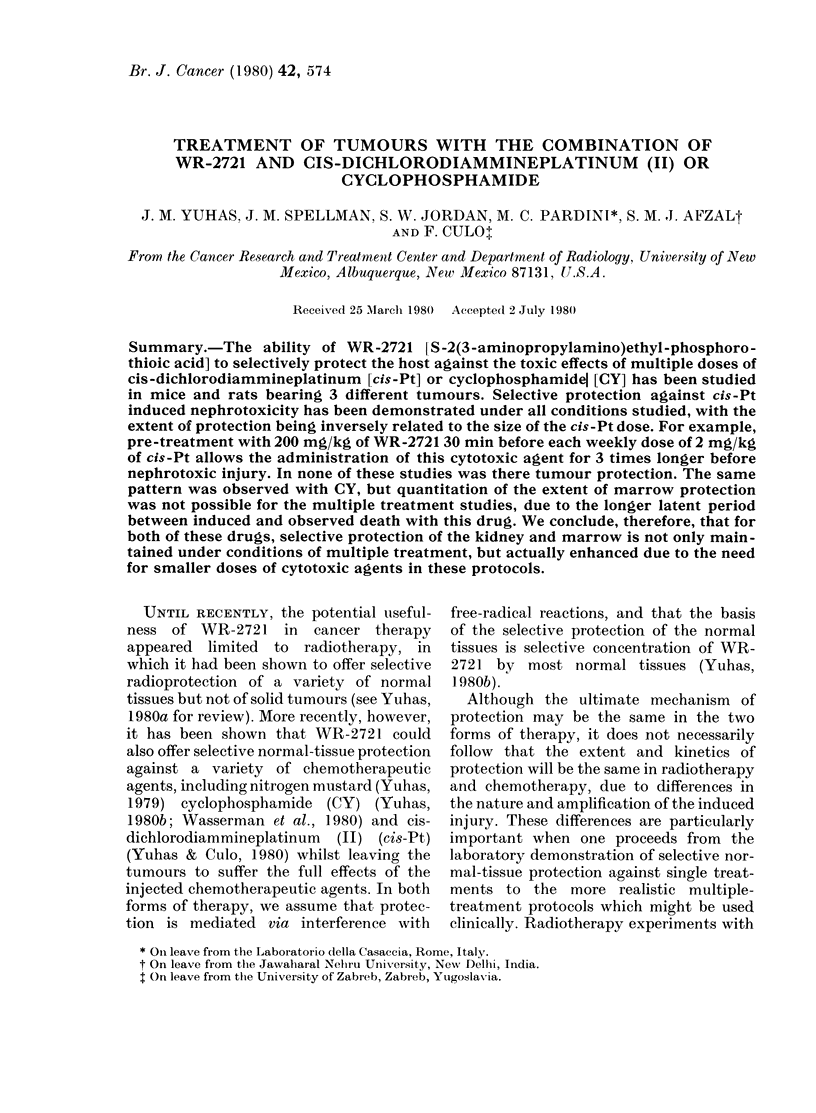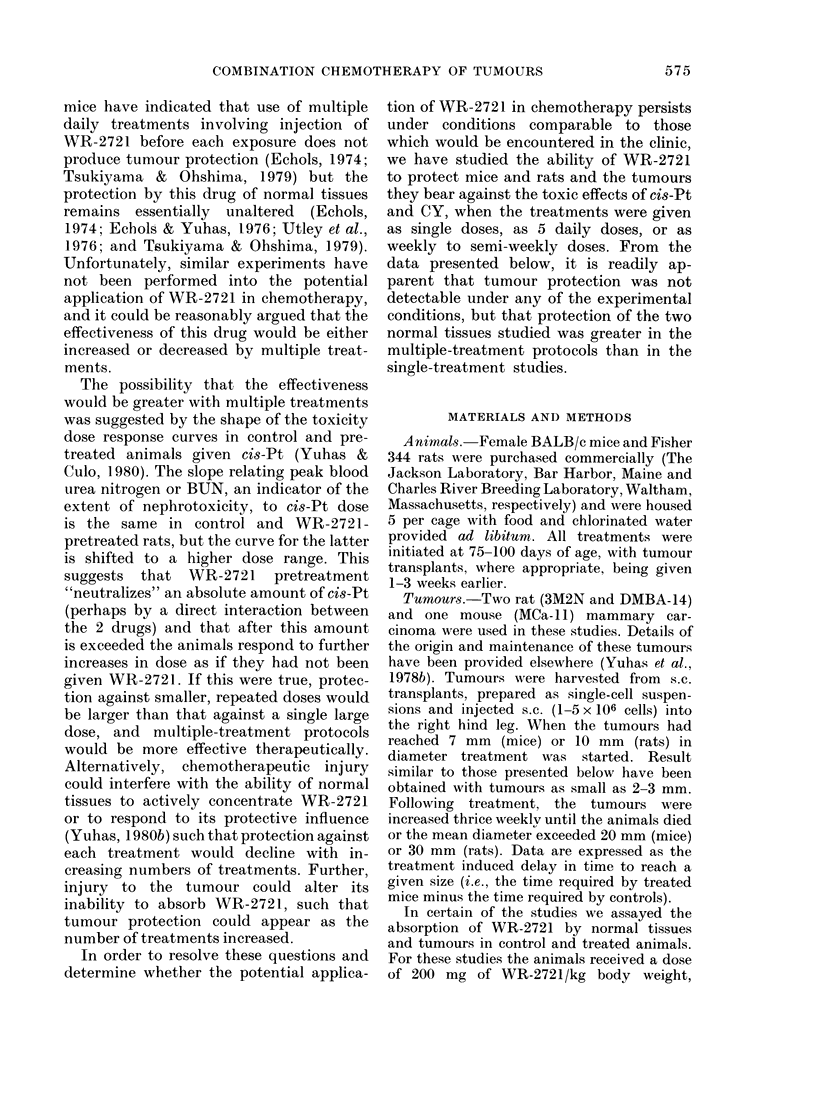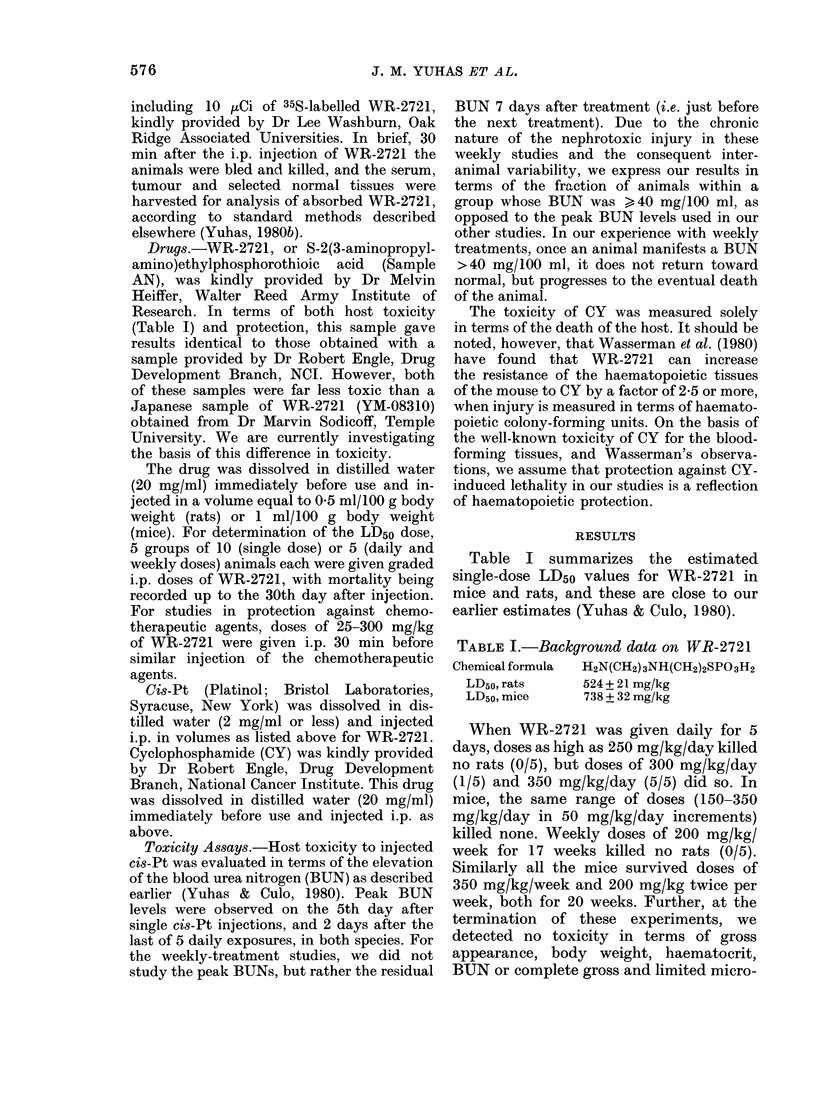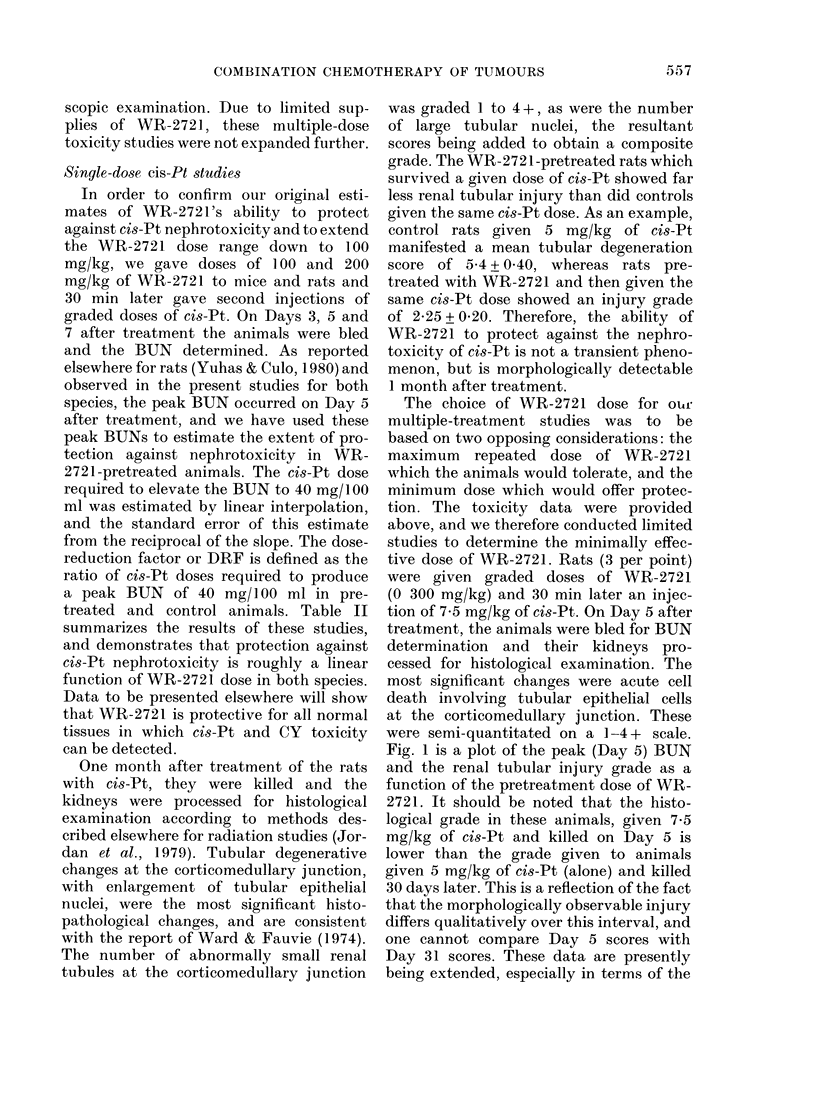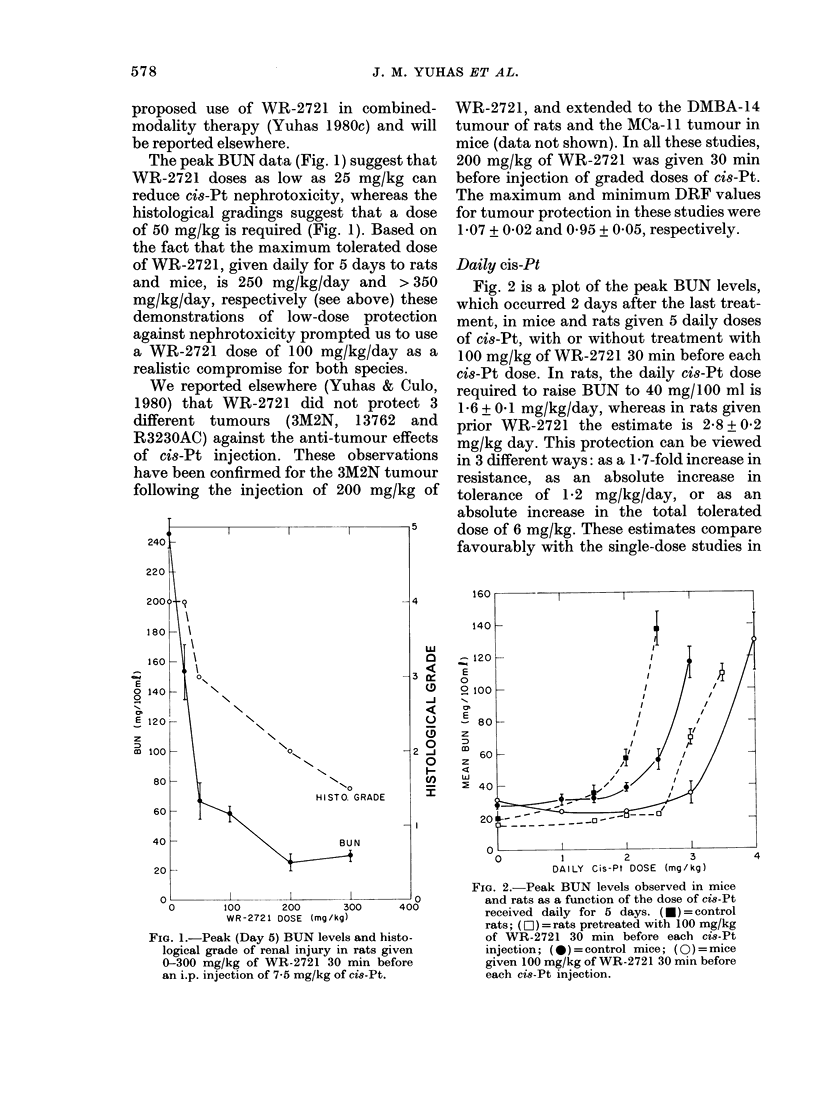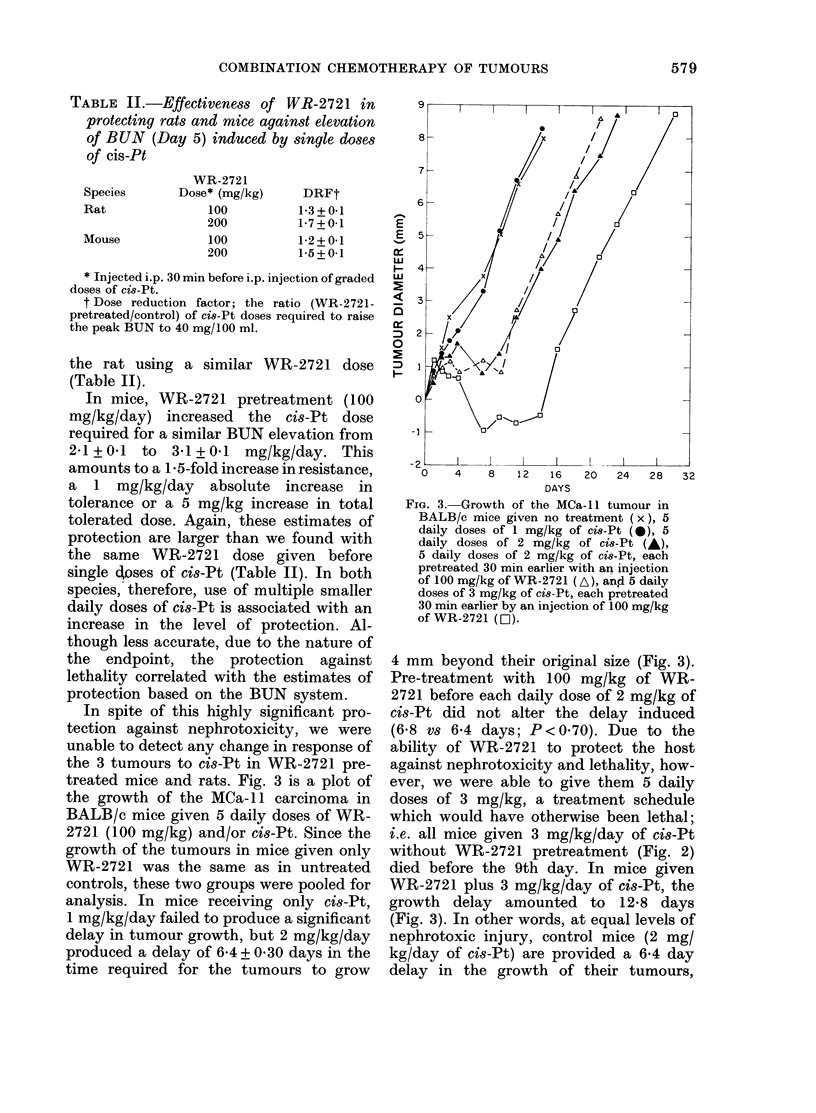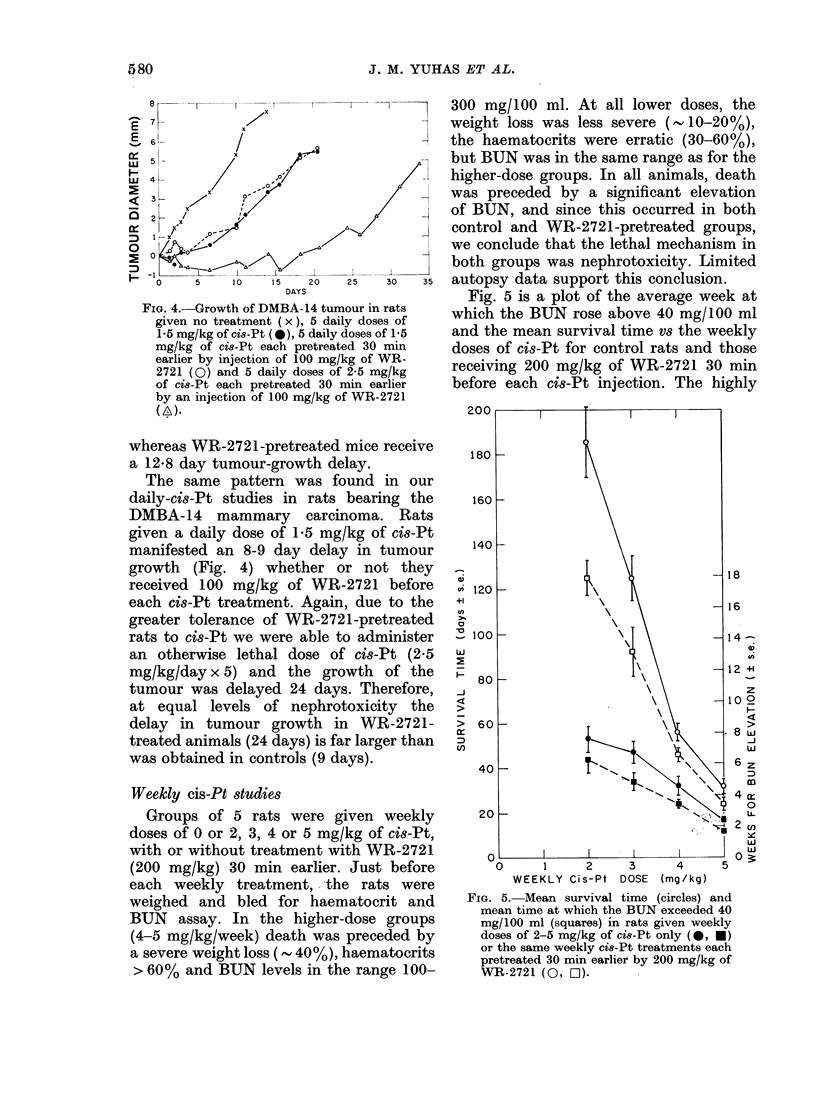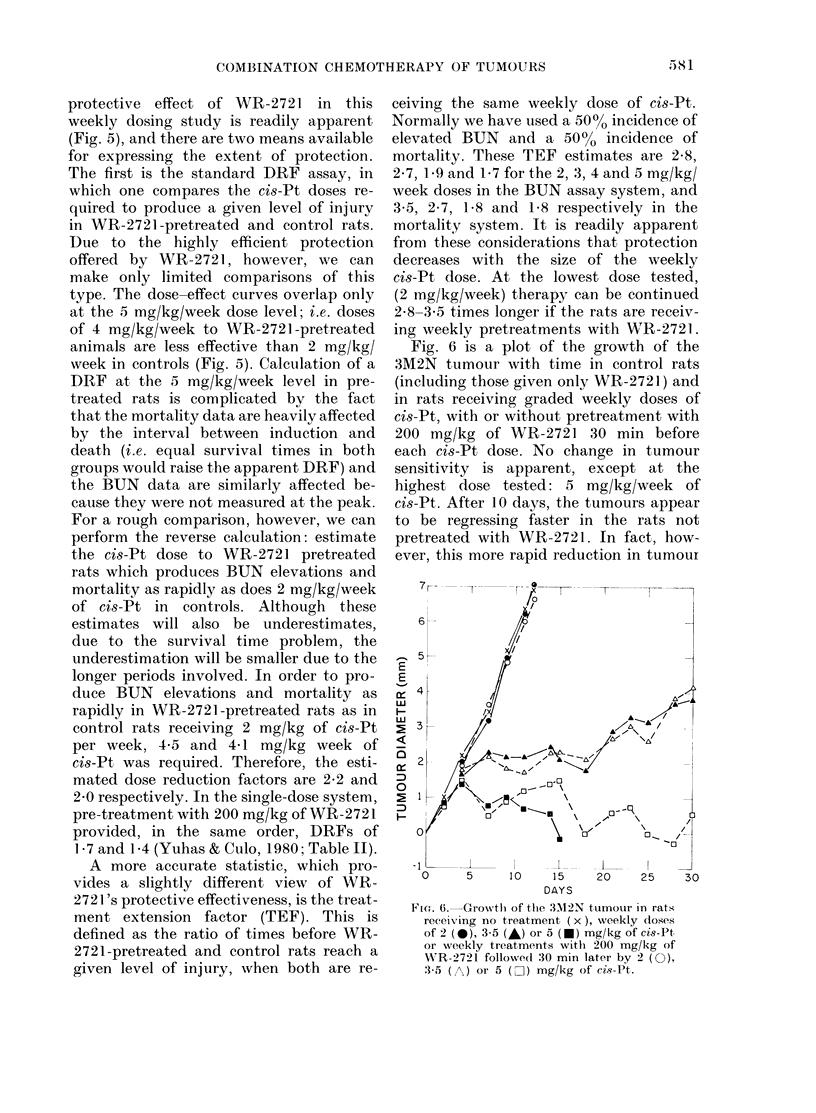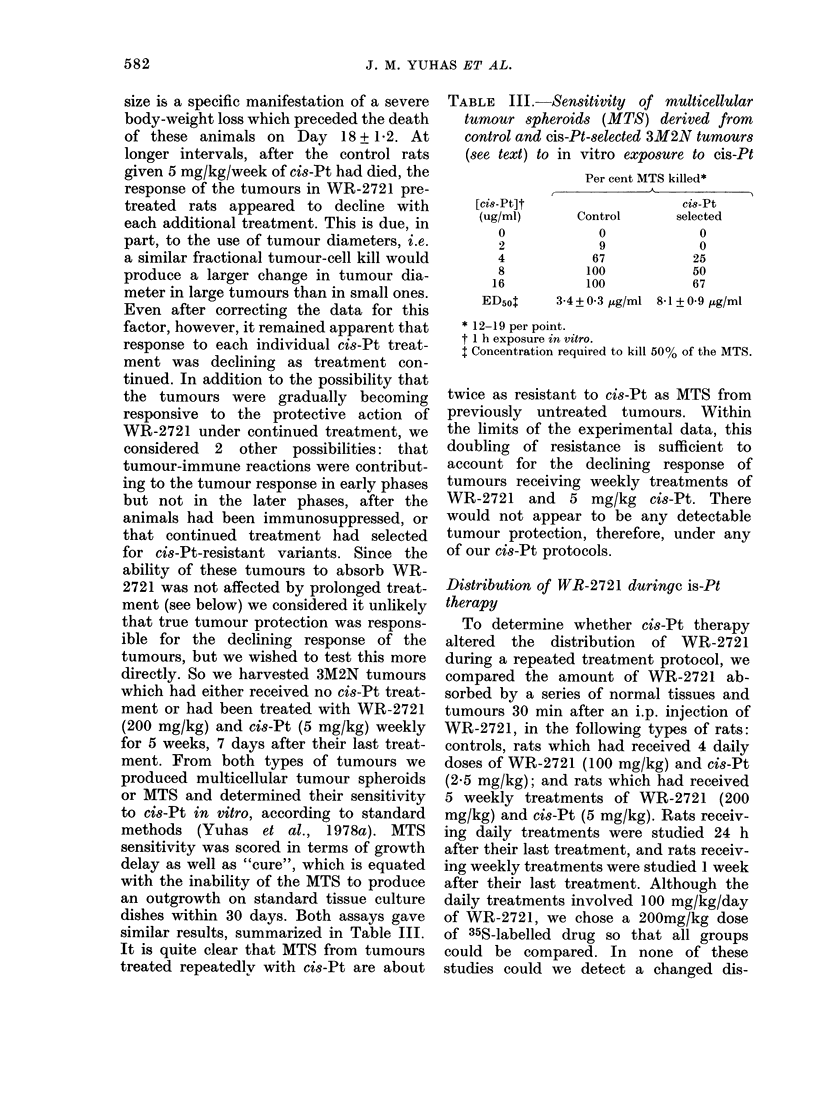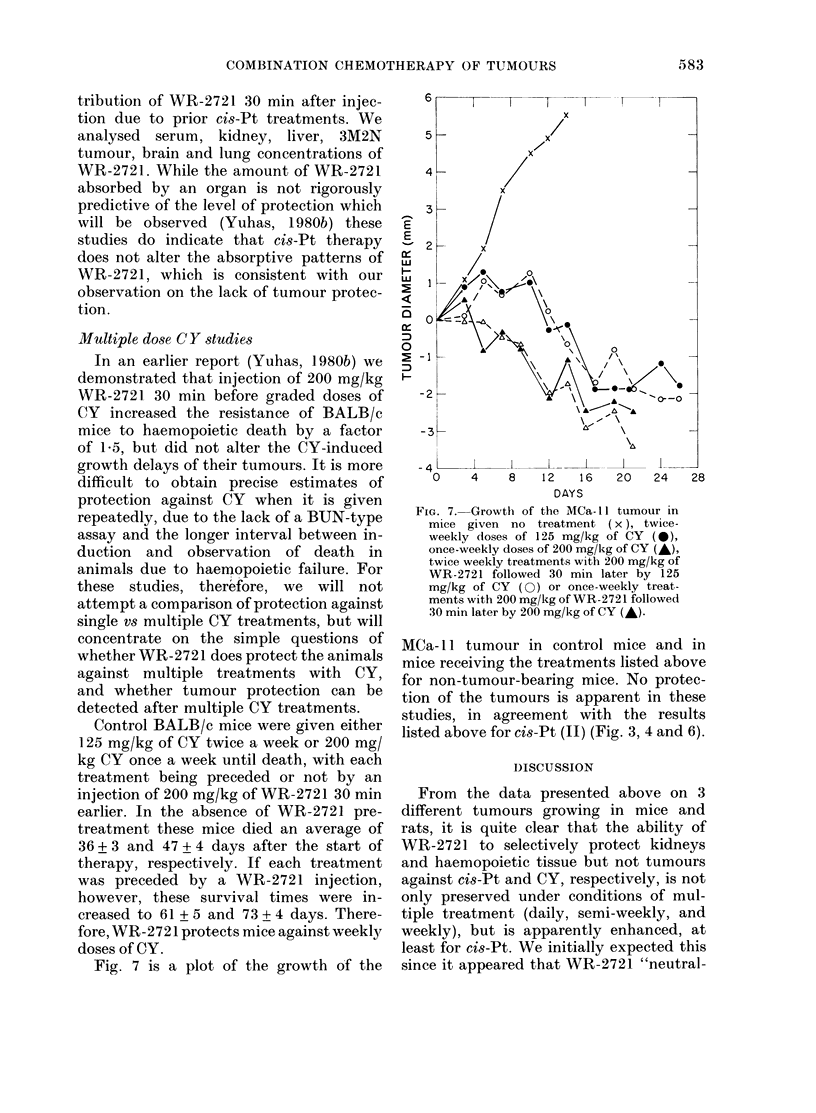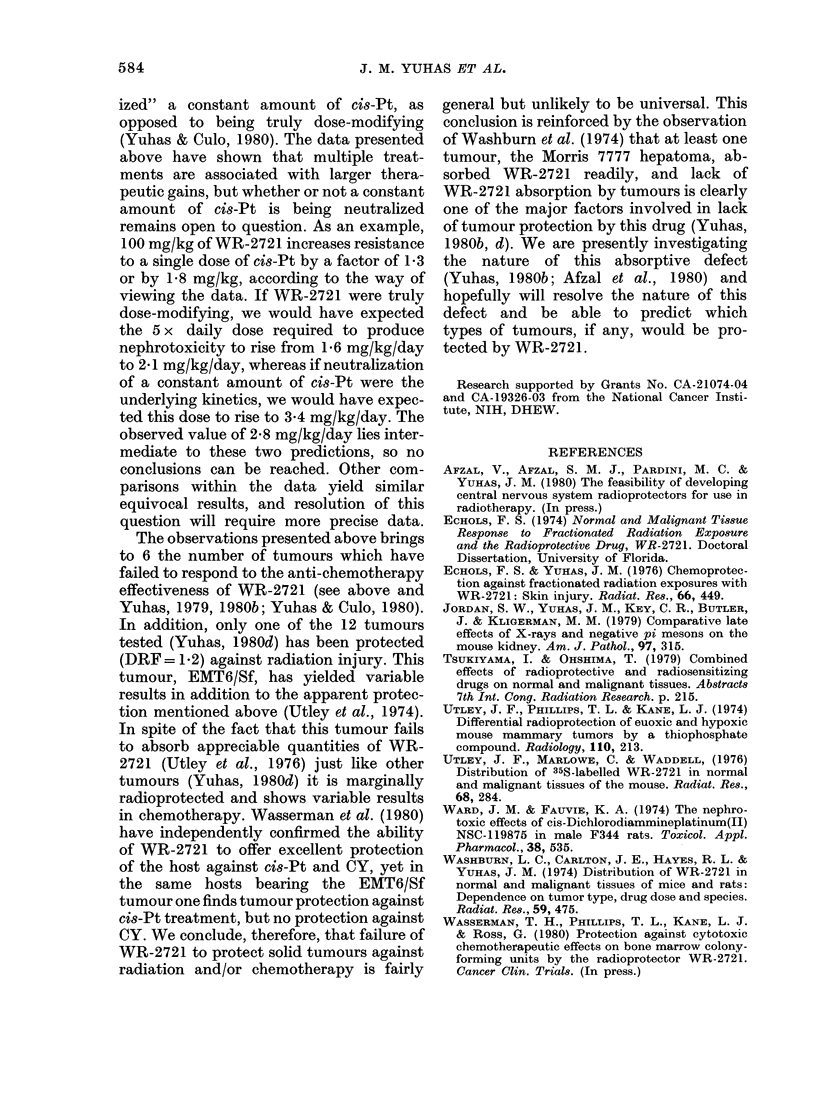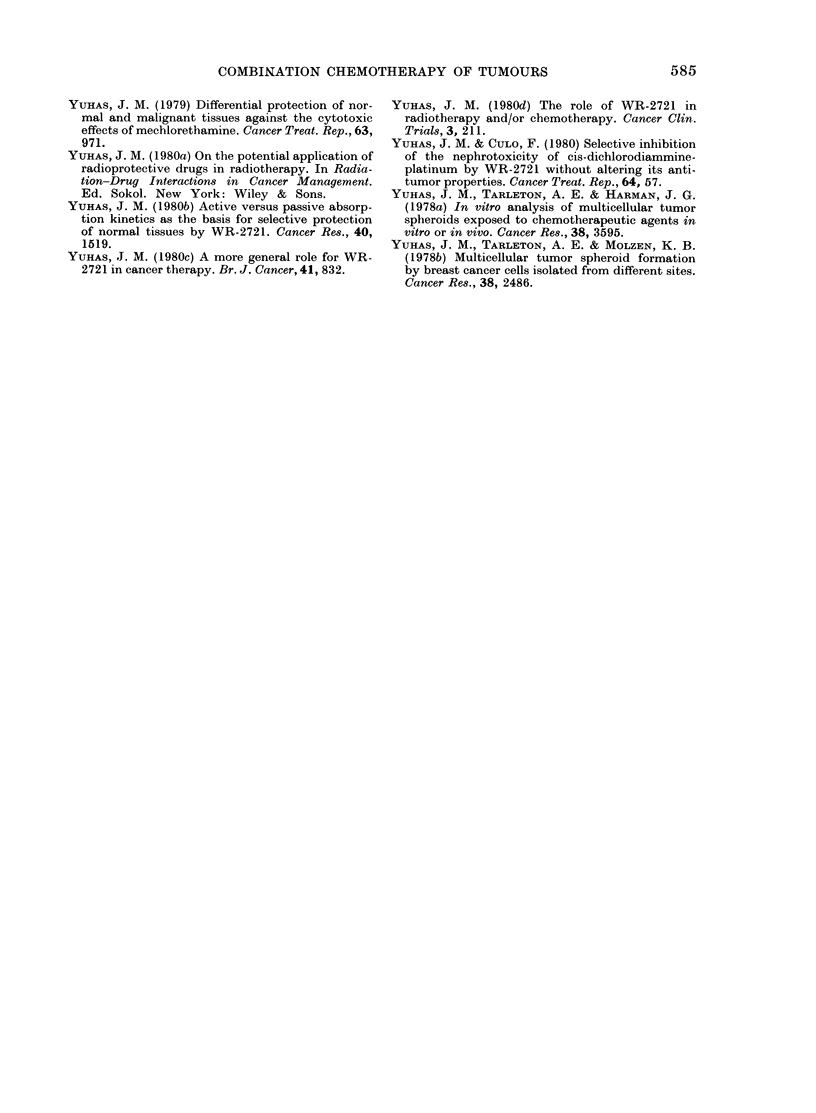# Treatment of tumours with the combination of WR-2721 and cis-dichlorodiammineplatinum (II) or cyclophosphamide.

**DOI:** 10.1038/bjc.1980.282

**Published:** 1980-10

**Authors:** J. M. Yuhas, J. M. Spellman, S. W. Jordan, M. C. Pardini, S. M. Afzal, F. Culo

## Abstract

The ability of WR-2721 [S-2(3-aminopropylamino)ethyl-phosporothioic acid] to selectively protect the host against the toxic effects of multiple doses of cis-dichlorodiammineplatinum [cis-Pt] or cyclophosphamide [CY] has been studied in mice and rats bearing 3 different tumours. Selective protection against cis-Pt induced nephrotoxicity has been demonstrated under all conditions studied, with the extent of protection being inversely related to the size of the cis-Pt dose. For example, pre-treatment with 200 mg/kg of WR-2721 30 min before each weekly dose of 2 mg/kg of cis-Pt allows the administration of this cytotoxic agent for 3 times longer before nephrotoxic injury. In none of these studies was there tumour protection. The same pattern was observed with CY, but quantitation of the extent of marrow protection was not possible for the multiple treatment studies, due to the longer latent period between induced and observed death with this drug. We conclude, therefore, that for both of these drugs, selective protection of the kidney and marrow is not only maintained under conditions of multiple treatment, but actually enhanced due to the need for smaller doses of cytotoxic agents in these protocols.


*Br. J. Cancer* (1980) **42**, 574

### TREATMENT OF TUMOURS WITH THE COMBINATION OF WR-2721 AND CIS-DICHLORODIAMMINEPLATINUM (II) OR CYCLOPHOSPHAMIDE

J. M. YUHAS, J. M. SPELLMAN, S. W. JORDAN, M. C. PARDINI*, S. M. J. AFZAL† AND F. CULO‡

*From the Cancer Research and Treatment Center and Department of Radiology, University of New Mexico, Albuquerque, New Mexico* 87131, *U.S.A.*

Received 25 March 1980 Accepted 2 July 1980

**Summary.—The ability of WR-2721 [S-2(3-aminopropylamino)ethyl-phosphorothioic acid] to selectively protect the host against the toxic effects of multiple doses of cis-dichlorodiammineplatinum [*cis*-Pt] or cyclophosphamide] [CY] has been studied in mice and rats bearing 3 different tumours. Selective protection against *cis*-Pt induced nephrotoxicity has been demonstrated under all conditions studied, with the extent of protection being inversely related to the size of the *cis*-Pt dose. For example, pre-treatment with 200 mg/kg of WR-2721 30 min before each weekly dose of 2 mg/kg of *cis*-Pt allows the administration of this cytotoxic agent for 3 times longer before nephrotoxic injury. In none of these studies was there tumour protection. The same pattern was observed with CY, but quantitation of the extent of marrow protection was not possible for the multiple treatment studies, due to the longer latent period between induced and observed death with this drug. We conclude, therefore, that for both of these drugs, selective protection of the kidney and marrow is not only maintained under conditions of multiple treatment, but actually enhanced due to the need for smaller doses of cytotoxic agents in these protocols.**

UNTIL RECENTLY, the potential usefulness of WR-2721 in cancer therapy appeared limited to radiotherapy, in which it had been shown to offer selective radioprotection of a variety of normal tissues but not of solid tumours (see Yuhas, 1980*a* for review). More recently, however, it has been shown that WR-2721 could also offer selective normal-tissue protection against a variety of chemotherapeutic agents, including nitrogen mustard (Yuhas, 1979) cyclophosphamide (CY) (Yuhas, 1980*b*; Wasserman *et al.*, 1980) and cis-dichlorodiammineplatinum (II) (*cis*-Pt) (Yuhas & Culo, 1980) whilst leaving the tumours to suffer the full effects of the injected chemotherapeutic agents. In both forms of therapy, we assume that protection is mediated *via* interference with free-radical reactions, and that the basis of the selective protection of the normal tissues is selective concentration of WR-2721 by most normal tissues (Yuhas, 1980*b*).

Although the ultimate mechanism of protection may be the same in the two forms of therapy, it does not necessarily follow that the extent and kinetics of protection will be the same in radiotherapy and chemotherapy, due to differences in the nature and amplification of the induced injury. These differences are particularly important when one proceeds from the laboratory demonstration of selective normal-tissue protection against single treatments to the more realistic multiple-treatment protocols which might be used clinically. Radiotherapy experiments with

* On leave from the Laboratorio della Casaccia, Rome, Italy.
† On leave from the Jawaharal Nehru University, New Delhi, India.
‡ On leave from the University of Zabreb, Zabreb, Yugoslavia.

mice have indicated that use of multiple daily treatments involving injection of WR-2721 before each exposure does not produce tumour protection (Echols, 1974; Tsukiyama & Ohshima, 1979) but the protection by this drug of normal tissues remains essentially unaltered (Echols, 1974; Echols & Yuhas, 1976; Utley *et al.*, 1976; and Tsukiyama & Ohshima, 1979). Unfortunately, similar experiments have not been performed into the potential application of WR-2721 in chemotherapy, and it could be reasonably argued that the effectiveness of this drug would be either increased or decreased by multiple treatments.

The possibility that the effectiveness would be greater with multiple treatments was suggested by the shape of the toxicity dose response curves in control and pretreated animals given *cis*-Pt (Yuhas & Culo, 1980). The slope relating peak blood urea nitrogen or BUN, an indicator of the extent of nephrotoxicity, to *cis*-Pt dose is the same in control and WR-2721-pretreated rats, but the curve for the latter is shifted to a higher dose range. This suggests that WR-2721 pretreatment "neutralizes" an absolute amount of *cis*-Pt (perhaps by a direct interaction between the 2 drugs) and that after this amount is exceeded the animals respond to further increases in dose as if they had not been given WR-2721. If this were true, protection against smaller, repeated doses would be larger than that against a single large dose, and multiple-treatment protocols would be more effective therapeutically. Alternatively, chemotherapeutic injury could interfere with the ability of normal tissues to actively concentrate WR-2721 or to respond to its protective influence (Yuhas, 1980*b*) such that protection against each treatment would decline with increasing numbers of treatments. Further, injury to the tumour could alter its inability to absorb WR-2721, such that tumour protection could appear as the number of treatments increased.

In order to resolve these questions and determine whether the potential application of WR-2721 in chemotherapy persists under conditions comparable to those which would be encountered in the clinic, we have studied the ability of WR-2721 to protect mice and rats and the tumours they bear against the toxic effects of *cis*-Pt and CY, when the treatments were given as single doses, as 5 daily doses, or as weekly to semi-weekly doses. From the data presented below, it is readily apparent that tumour protection was not detectable under any of the experimental conditions, but that protection of the two normal tissues studied was greater in the multiple-treatment protocols than in the single-treatment studies.

### MATERIALS AND METHODS

*Animals.*—Female BALB/c mice and Fisher 344 rats were purchased commercially (The Jackson Laboratory, Bar Harbor, Maine and Charles River Breeding Laboratory, Waltham, Massachusetts, respectively) and were housed 5 per cage with food and chlorinated water provided *ad libitum*. All treatments were initiated at 75–100 days of age, with tumour transplants, where appropriate, being given 1–3 weeks earlier.

*Tumours.*—Two rat (3M2N and DMBA-14) and one mouse (MCa-11) mammary carcinoma were used in these studies. Details of the origin and maintenance of these tumours have been provided elsewhere (Yuhas *et al.*, 1978*b*). Tumours were harvested from s.c. transplants, prepared as single-cell suspensions and injected s.c. ($1\text{–}5 \times 10^6$ cells) into the right hind leg. When the tumours had reached 7 mm (mice) or 10 mm (rats) in diameter treatment was started. Result similar to those presented below have been obtained with tumours as small as 2–3 mm. Following treatment, the tumours were increased thrice weekly until the animals died or the mean diameter exceeded 20 mm (mice) or 30 mm (rats). Data are expressed as the treatment induced delay in time to reach a given size (*i.e.*, the time required by treated mice minus the time required by controls).

In certain of the studies we assayed the absorption of WR-2721 by normal tissues and tumours in control and treated animals. For these studies the animals received a dose of 200 mg of WR-2721/kg body weight, including 10 μCi of $^{35}S$-labelled WR-2721, kindly provided by Dr Lee Washburn, Oak Ridge Associated Universities. In brief, 30 min after the i.p. injection of WR-2721 the animals were bled and killed, and the serum, tumour and selected normal tissues were harvested for analysis of absorbed WR-2721, according to standard methods described elsewhere (Yuhas, 1980*b*).

*Drugs.*—WR-2721, or S-2(3-aminopropylamino)ethylphosphorothioic acid (Sample AN), was kindly provided by Dr Melvin Heiffer, Walter Reed Army Institute of Research. In terms of both host toxicity (Table I) and protection, this sample gave results identical to those obtained with a sample provided by Dr Robert Engle, Drug Development Branch, NCI. However, both of these samples were far less toxic than a Japanese sample of WR-2721 (YM-08310) obtained from Dr Marvin Sodicoff, Temple University. We are currently investigating the basis of this difference in toxicity.

The drug was dissolved in distilled water (20 mg/ml) immediately before use and injected in a volume equal to 0·5 ml/100 g body weight (rats) or 1 ml/100 g body weight (mice). For determination of the $LD_{50}$ dose, 5 groups of 10 (single dose) or 5 (daily and weekly doses) animals each were given graded i.p. doses of WR-2721, with mortality being recorded up to the 30th day after injection. For studies in protection against chemotherapeutic agents, doses of 25–300 mg/kg of WR-2721 were given i.p. 30 min before similar injection of the chemotherapeutic agents.

*Cis*-Pt (Platinol; Bristol Laboratories, Syracuse, New York) was dissolved in distilled water (2 mg/ml or less) and injected i.p. in volumes as listed above for WR-2721. Cyclophosphamide (CY) was kindly provided by Dr Robert Engle, Drug Development Branch, National Cancer Institute. This drug was dissolved in distilled water (20 mg/ml) immediately before use and injected i.p. as above.

*Toxicity Assays.*—Host toxicity to injected *cis*-Pt was evaluated in terms of the elevation of the blood urea nitrogen (BUN) as described earlier (Yuhas & Culo, 1980). Peak BUN levels were observed on the 5th day after single *cis*-Pt injections, and 2 days after the last of 5 daily exposures, in both species. For the weekly-treatment studies, we did not study the peak BUNs, but rather the residual BUN 7 days after treatment (*i.e.* just before the next treatment). Due to the chronic nature of the nephrotoxic injury in these weekly studies and the consequent inter-animal variability, we express our results in terms of the fraction of animals within a group whose BUN was ≥40 mg/100 ml, as opposed to the peak BUN levels used in our other studies. In our experience with weekly treatments, once an animal manifests a BUN >40 mg/100 ml, it does not return toward normal, but progresses to the eventual death of the animal.

The toxicity of CY was measured solely in terms of the death of the host. It should be noted, however, that Wasserman *et al.* (1980) have found that WR-2721 can increase the resistance of the haematopoietic tissues of the mouse to CY by a factor of 2·5 or more, when injury is measured in terms of haematopoietic colony-forming units. On the basis of the well-known toxicity of CY for the blood-forming tissues, and Wasserman's observations, we assume that protection against CY-induced lethality in our studies is a reflection of haematopoietic protection.

### RESULTS

Table I summarizes the estimated single-dose $LD_{50}$ values for WR-2721 in mice and rats, and these are close to our earlier estimates (Yuhas & Culo, 1980).

TABLE I.—*Background data on WR*-2721

| | |
|---|---|
| Chemical formula | $H_2N(CH_2)_3NH(CH_2)_2SPO_3H_2$ |
| $LD_{50}$, rats | 524 ± 21 mg/kg |
| $LD_{50}$, mice | 738 ± 32 mg/kg |

When WR-2721 was given daily for 5 days, doses as high as 250 mg/kg/day killed no rats (0/5), but doses of 300 mg/kg/day (1/5) and 350 mg/kg/day (5/5) did so. In mice, the same range of doses (150–350 mg/kg/day in 50 mg/kg/day increments) killed none. Weekly doses of 200 mg/kg/week for 17 weeks killed no rats (0/5). Similarly all the mice survived doses of 350 mg/kg/week and 200 mg/kg twice per week, both for 20 weeks. Further, at the termination of these experiments, we detected no toxicity in terms of gross appearance, body weight, haematocrit, BUN or complete gross and limited microscopic examination. Due to limited supplies of WR-2721, these multiple-dose toxicity studies were not expanded further.

*Single-dose* cis-*Pt studies*

In order to confirm our original estimates of WR-2721's ability to protect against *cis*-Pt nephrotoxicity and to extend the WR-2721 dose range down to 100 mg/kg, we gave doses of 100 and 200 mg/kg of WR-2721 to mice and rats and 30 min later gave second injections of graded doses of *cis*-Pt. On Days 3, 5 and 7 after treatment the animals were bled and the BUN determined. As reported elsewhere for rats (Yuhas & Culo, 1980) and observed in the present studies for both species, the peak BUN occurred on Day 5 after treatment, and we have used these peak BUNs to estimate the extent of protection against nephrotoxicity in WR-2721-pretreated animals. The *cis*-Pt dose required to elevate the BUN to 40 mg/100 ml was estimated by linear interpolation, and the standard error of this estimate from the reciprocal of the slope. The dose-reduction factor or DRF is defined as the ratio of *cis*-Pt doses required to produce a peak BUN of 40 mg/100 ml in pretreated and control animals. Table II summarizes the results of these studies, and demonstrates that protection against *cis*-Pt nephrotoxicity is roughly a linear function of WR-2721 dose in both species. Data to be presented elsewhere will show that WR-2721 is protective for all normal tissues in which *cis*-Pt and CY toxicity can be detected.

One month after treatment of the rats with *cis*-Pt, they were killed and the kidneys were processed for histological examination according to methods described elsewhere for radiation studies (Jordan *et al.*, 1979). Tubular degenerative changes at the corticomedullary junction, with enlargement of tubular epithelial nuclei, were the most significant histopathological changes, and are consistent with the report of Ward & Fauvie (1974). The number of abnormally small renal tubules at the corticomedullary junction was graded 1 to 4+, as were the number of large tubular nuclei, the resultant scores being added to obtain a composite grade. The WR-2721-pretreated rats which survived a given dose of *cis*-Pt showed far less renal tubular injury than did controls given the same *cis*-Pt dose. As an example, control rats given 5 mg/kg of *cis*-Pt manifested a mean tubular degeneration score of $5{\cdot}4 \pm 0{\cdot}40$, whereas rats pretreated with WR-2721 and then given the same *cis*-Pt dose showed an injury grade of $2{\cdot}25 \pm 0{\cdot}20$. Therefore, the ability of WR-2721 to protect against the nephrotoxicity of *cis*-Pt is not a transient phenomenon, but is morphologically detectable 1 month after treatment.

The choice of WR-2721 dose for our multiple-treatment studies was to be based on two opposing considerations: the maximum repeated dose of WR-2721 which the animals would tolerate, and the minimum dose which would offer protection. The toxicity data were provided above, and we therefore conducted limited studies to determine the minimally effective dose of WR-2721. Rats (3 per point) were given graded doses of WR-2721 (0 300 mg/kg) and 30 min later an injection of 7·5 mg/kg of *cis*-Pt. On Day 5 after treatment, the animals were bled for BUN determination and their kidneys processed for histological examination. The most significant changes were acute cell death involving tubular epithelial cells at the corticomedullary junction. These were semi-quantitated on a 1–4+ scale. Fig. 1 is a plot of the peak (Day 5) BUN and the renal tubular injury grade as a function of the pretreatment dose of WR-2721. It should be noted that the histological grade in these animals, given 7·5 mg/kg of *cis*-Pt and killed on Day 5 is lower than the grade given to animals given 5 mg/kg of *cis*-Pt (alone) and killed 30 days later. This is a reflection of the fact that the morphologically observable injury differs qualitatively over this interval, and one cannot compare Day 5 scores with Day 31 scores. These data are presently being extended, especially in terms of the proposed use of WR-2721 in combined-modality therapy (Yuhas 1980*c*) and will be reported elsewhere.

The peak BUN data (Fig. 1) suggest that WR-2721 doses as low as 25 mg/kg can reduce *cis*-Pt nephrotoxicity, whereas the histological gradings suggest that a dose of 50 mg/kg is required (Fig. 1). Based on the fact that the maximum tolerated dose of WR-2721, given daily for 5 days to rats and mice, is 250 mg/kg/day and >350 mg/kg/day, respectively (see above) these demonstrations of low-dose protection against nephrotoxicity prompted us to use a WR-2721 dose of 100 mg/kg/day as a realistic compromise for both species.

We reported elsewhere (Yuhas & Culo, 1980) that WR-2721 did not protect 3 different tumours (3M2N, 13762 and R3230AC) against the anti-tumour effects of *cis*-Pt injection. These observations have been confirmed for the 3M2N tumour following the injection of 200 mg/kg of WR-2721, and extended to the DMBA-14 tumour of rats and the MCa-11 tumour in mice (data not shown). In all these studies, 200 mg/kg of WR-2721 was given 30 min before injection of graded doses of *cis*-Pt. The maximum and minimum DRF values for tumour protection in these studies were 1·07 ± 0·02 and 0·95 ± 0·05, respectively.

FIG. 1.—Peak (Day 5) BUN levels and histological grade of renal injury in rats given 0–300 mg/kg of WR-2721 30 min before an i.p. injection of 7·5 mg/kg of *cis*-Pt.

*Daily* cis-*Pt*

Fig. 2 is a plot of the peak BUN levels, which occurred 2 days after the last treatment, in mice and rats given 5 daily doses of *cis*-Pt, with or without treatment with 100 mg/kg of WR-2721 30 min before each *cis*-Pt dose. In rats, the daily *cis*-Pt dose required to raise BUN to 40 mg/100 ml is 1·6 ± 0·1 mg/kg/day, whereas in rats given prior WR-2721 the estimate is 2·8 ± 0·2 mg/kg day. This protection can be viewed in 3 different ways: as a 1·7-fold increase in resistance, as an absolute increase in tolerance of 1·2 mg/kg/day, or as an absolute increase in the total tolerated dose of 6 mg/kg. These estimates compare favourably with the single-dose studies in

FIG. 2.—Peak BUN levels observed in mice and rats as a function of the dose of *cis*-Pt received daily for 5 days. (■) = control rats; (□) = rats pretreated with 100 mg/kg of WR-2721 30 min before each *cis*-Pt injection; (●) = control mice; (○) = mice given 100 mg/kg of WR-2721 30 min before each *cis*-Pt injection.

TABLE II.—*Effectiveness of WR-2721 in protecting rats and mice against elevation of BUN (Day 5) induced by single doses of* cis-*Pt*

| Species | WR-2721 Dose* (mg/kg) | DRF† |
|---|---|---|
| Rat | 100 | 1·3 ± 0·1 |
| | 200 | 1·7 ± 0·1 |
| Mouse | 100 | 1·2 ± 0·1 |
| | 200 | 1·5 ± 0·1 |

* Injected i.p. 30 min before i.p. injection of graded doses of *cis*-Pt.

† Dose reduction factor; the ratio (WR-2721-pretreated/control) of *cis*-Pt doses required to raise the peak BUN to 40 mg/100 ml.

the rat using a similar WR-2721 dose (Table II).

In mice, WR-2721 pretreatment (100 mg/kg/day) increased the *cis*-Pt dose required for a similar BUN elevation from 2·1 ± 0·1 to 3·1 ± 0·1 mg/kg/day. This amounts to a 1·5-fold increase in resistance, a 1 mg/kg/day absolute increase in tolerance or a 5 mg/kg increase in total tolerated dose. Again, these estimates of protection are larger than we found with the same WR-2721 dose given before single doses of *cis*-Pt (Table II). In both species, therefore, use of multiple smaller daily doses of *cis*-Pt is associated with an increase in the level of protection. Although less accurate, due to the nature of the endpoint, the protection against lethality correlated with the estimates of protection based on the BUN system.

In spite of this highly significant protection against nephrotoxicity, we were unable to detect any change in response of the 3 tumours to *cis*-Pt in WR-2721 pretreated mice and rats. Fig. 3 is a plot of the growth of the MCa-11 carcinoma in BALB/c mice given 5 daily doses of WR-2721 (100 mg/kg) and/or *cis*-Pt. Since the growth of the tumours in mice given only WR-2721 was the same as in untreated controls, these two groups were pooled for analysis. In mice receiving only *cis*-Pt, 1 mg/kg/day failed to produce a significant delay in tumour growth, but 2 mg/kg/day produced a delay of 6·4 ± 0·30 days in the time required for the tumours to grow

FIG. 3.—Growth of the MCa-11 tumour in BALB/c mice given no treatment (×), 5 daily doses of 1 mg/kg of *cis*-Pt (●), 5 daily doses of 2 mg/kg of *cis*-Pt (▲), 5 daily doses of 2 mg/kg of *cis*-Pt, each pretreated 30 min earlier with an injection of 100 mg/kg of WR-2721 (△), and 5 daily doses of 3 mg/kg of *cis*-Pt, each pretreated 30 min earlier by an injection of 100 mg/kg of WR-2721 (□).

4 mm beyond their original size (Fig. 3). Pre-treatment with 100 mg/kg of WR-2721 before each daily dose of 2 mg/kg of *cis*-Pt did not alter the delay induced (6·8 *vs* 6·4 days; $P < 0·70$). Due to the ability of WR-2721 to protect the host against nephrotoxicity and lethality, however, we were able to give them 5 daily doses of 3 mg/kg, a treatment schedule which would have otherwise been lethal; *i.e.* all mice given 3 mg/kg/day of *cis*-Pt without WR-2721 pretreatment (Fig. 2) died before the 9th day. In mice given WR-2721 plus 3 mg/kg/day of *cis*-Pt, the growth delay amounted to 12·8 days (Fig. 3). In other words, at equal levels of nephrotoxic injury, control mice (2 mg/kg/day of *cis*-Pt) are provided a 6·4 day delay in the growth of their tumours,

FIG. 4.—Growth of DMBA-14 tumour in rats given no treatment (×), 5 daily doses of 1·5 mg/kg of *cis*-Pt (●), 5 daily doses of 1·5 mg/kg of *cis*-Pt each pretreated 30 min earlier by injection of 100 mg/kg of WR-2721 (○) and 5 daily doses of 2·5 mg/kg of *cis*-Pt each pretreated 30 min earlier by an injection of 100 mg/kg of WR-2721 (△).

whereas WR-2721-pretreated mice receive a 12·8 day tumour-growth delay.

The same pattern was found in our daily-*cis*-Pt studies in rats bearing the DMBA-14 mammary carcinoma. Rats given a daily dose of 1·5 mg/kg of *cis*-Pt manifested an 8-9 day delay in tumour growth (Fig. 4) whether or not they received 100 mg/kg of WR-2721 before each *cis*-Pt treatment. Again, due to the greater tolerance of WR-2721-pretreated rats to *cis*-Pt we were able to administer an otherwise lethal dose of *cis*-Pt (2·5 mg/kg/day × 5) and the growth of the tumour was delayed 24 days. Therefore, at equal levels of nephrotoxicity the delay in tumour growth in WR-2721-treated animals (24 days) is far larger than was obtained in controls (9 days).

### *Weekly* cis-*Pt studies*

Groups of 5 rats were given weekly doses of 0 or 2, 3, 4 or 5 mg/kg of *cis*-Pt, with or without treatment with WR-2721 (200 mg/kg) 30 min earlier. Just before each weekly treatment, the rats were weighed and bled for haematocrit and BUN assay. In the higher-dose groups (4–5 mg/kg/week) death was preceded by a severe weight loss (~40%), haematocrits >60% and BUN levels in the range 100–300 mg/100 ml. At all lower doses, the weight loss was less severe (~10–20%), the haematocrits were erratic (30–60%), but BUN was in the same range as for the higher-dose groups. In all animals, death was preceded by a significant elevation of BUN, and since this occurred in both control and WR-2721-pretreated groups, we conclude that the lethal mechanism in both groups was nephrotoxicity. Limited autopsy data support this conclusion.

Fig. 5 is a plot of the average week at which the BUN rose above 40 mg/100 ml and the mean survival time *vs* the weekly doses of *cis*-Pt for control rats and those receiving 200 mg/kg of WR-2721 30 min before each *cis*-Pt injection. The highly

FIG. 5.—Mean survival time (circles) and mean time at which the BUN exceeded 40 mg/100 ml (squares) in rats given weekly doses of 2–5 mg/kg of *cis*-Pt only (●, ■) or the same weekly *cis*-Pt treatments each pretreated 30 min earlier by 200 mg/kg of WR-2721 (○, □).

protective effect of WR-2721 in this weekly dosing study is readily apparent (Fig. 5), and there are two means available for expressing the extent of protection. The first is the standard DRF assay, in which one compares the *cis*-Pt doses required to produce a given level of injury in WR-2721-pretreated and control rats. Due to the highly efficient protection offered by WR-2721, however, we can make only limited comparisons of this type. The dose–effect curves overlap only at the 5 mg/kg/week dose level; *i.e.* doses of 4 mg/kg/week to WR-2721-pretreated animals are less effective than 2 mg/kg/week in controls (Fig. 5). Calculation of a DRF at the 5 mg/kg/week level in pretreated rats is complicated by the fact that the mortality data are heavily affected by the interval between induction and death (*i.e.* equal survival times in both groups would raise the apparent DRF) and the BUN data are similarly affected because they were not measured at the peak. For a rough comparison, however, we can perform the reverse calculation: estimate the *cis*-Pt dose to WR-2721 pretreated rats which produces BUN elevations and mortality as rapidly as does 2 mg/kg/week of *cis*-Pt in controls. Although these estimates will also be underestimates, due to the survival time problem, the underestimation will be smaller due to the longer periods involved. In order to produce BUN elevations and mortality as rapidly in WR-2721-pretreated rats as in control rats receiving 2 mg/kg of *cis*-Pt per week, 4·5 and 4·1 mg/kg week of *cis*-Pt was required. Therefore, the estimated dose reduction factors are 2·2 and 2·0 respectively. In the single-dose system, pre-treatment with 200 mg/kg of WR-2721 provided, in the same order, DRFs of 1·7 and 1·4 (Yuhas & Culo, 1980; Table II).

A more accurate statistic, which provides a slightly different view of WR-2721's protective effectiveness, is the treatment extension factor (TEF). This is defined as the ratio of times before WR-2721-pretreated and control rats reach a given level of injury, when both are receiving the same weekly dose of *cis*-Pt. Normally we have used a 50% incidence of elevated BUN and a 50% incidence of mortality. These TEF estimates are 2·8, 2·7, 1·9 and 1·7 for the 2, 3, 4 and 5 mg/kg/week doses in the BUN assay system, and 3·5, 2·7, 1·8 and 1·8 respectively in the mortality system. It is readily apparent from these considerations that protection decreases with the size of the weekly *cis*-Pt dose. At the lowest dose tested, (2 mg/kg/week) therapy can be continued 2·8–3·5 times longer if the rats are receiving weekly pretreatments with WR-2721.

Fig. 6 is a plot of the growth of the 3M2N tumour with time in control rats (including those given only WR-2721) and in rats receiving graded weekly doses of *cis*-Pt, with or without pretreatment with 200 mg/kg of WR-2721 30 min before each *cis*-Pt dose. No change in tumour sensitivity is apparent, except at the highest dose tested: 5 mg/kg/week of *cis*-Pt. After 10 days, the tumours appear to be regressing faster in the rats not pretreated with WR-2721. In fact, however, this more rapid reduction in tumour

FIG. 6.—Growth of the 3M2N tumour in rats receiving no treatment (×), weekly doses of 2 (●), 3·5 (▲) or 5 (■) mg/kg of *cis*-Pt or weekly treatments with 200 mg/kg of WR-2721 followed 30 min later by 2 (○), 3·5 (△) or 5 (□) mg/kg of *cis*-Pt.

size is a specific manifestation of a severe body-weight loss which preceded the death of these animals on Day $18 \pm 1{\cdot}2$. At longer intervals, after the control rats given 5 mg/kg/week of *cis*-Pt had died, the response of the tumours in WR-2721 pretreated rats appeared to decline with each additional treatment. This is due, in part, to the use of tumour diameters, *i.e.* a similar fractional tumour-cell kill would produce a larger change in tumour diameter in large tumours than in small ones. Even after correcting the data for this factor, however, it remained apparent that response to each individual *cis*-Pt treatment was declining as treatment continued. In addition to the possibility that the tumours were gradually becoming responsive to the protective action of WR-2721 under continued treatment, we considered 2 other possibilities: that tumour-immune reactions were contributing to the tumour response in early phases but not in the later phases, after the animals had been immunosuppressed, or that continued treatment had selected for *cis*-Pt-resistant variants. Since the ability of these tumours to absorb WR-2721 was not affected by prolonged treatment (see below) we considered it unlikely that true tumour protection was responsible for the declining response of the tumours, but we wished to test this more directly. So we harvested 3M2N tumours which had either received no *cis*-Pt treatment or had been treated with WR-2721 (200 mg/kg) and *cis*-Pt (5 mg/kg) weekly for 5 weeks, 7 days after their last treatment. From both types of tumours we produced multicellular tumour spheroids or MTS and determined their sensitivity to *cis*-Pt *in vitro*, according to standard methods (Yuhas *et al.*, 1978*a*). MTS sensitivity was scored in terms of growth delay as well as "cure", which is equated with the inability of the MTS to produce an outgrowth on standard tissue culture dishes within 30 days. Both assays gave similar results, summarized in Table III. It is quite clear that MTS from tumours treated repeatedly with *cis*-Pt are about twice as resistant to *cis*-Pt as MTS from previously untreated tumours. Within the limits of the experimental data, this doubling of resistance is sufficient to account for the declining response of tumours receiving weekly treatments of WR-2721 and 5 mg/kg *cis*-Pt. There would not appear to be any detectable tumour protection, therefore, under any of our *cis*-Pt protocols.

TABLE III.—*Sensitivity of multicellular tumour spheroids (MTS) derived from control and* cis-*Pt-selected 3M2N tumours (see text) to* in vitro *exposure to* cis-*Pt*

| [*cis*-Pt]† (ug/ml) | Per cent MTS killed* Control | *cis*-Pt selected |
|---|---|---|
| 0 | 0 | 0 |
| 2 | 9 | 0 |
| 4 | 67 | 25 |
| 8 | 100 | 50 |
| 16 | 100 | 67 |
| $ED_{50}$‡ | $3{\cdot}4 \pm 0{\cdot}3$ μg/ml | $8{\cdot}1 \pm 0{\cdot}9$ μg/ml |

* 12–19 per point.

† 1 h exposure *in vitro*.

‡ Concentration required to kill 50% of the MTS.

*Distribution of WR-2721 duringc is-Pt therapy*

To determine whether *cis*-Pt therapy altered the distribution of WR-2721 during a repeated treatment protocol, we compared the amount of WR-2721 absorbed by a series of normal tissues and tumours 30 min after an i.p. injection of WR-2721, in the following types of rats: controls, rats which had received 4 daily doses of WR-2721 (100 mg/kg) and *cis*-Pt (2·5 mg/kg); and rats which had received 5 weekly treatments of WR-2721 (200 mg/kg) and *cis*-Pt (5 mg/kg). Rats receiving daily treatments were studied 24 h after their last treatment, and rats receiving weekly treatments were studied 1 week after their last treatment. Although the daily treatments involved 100 mg/kg/day of WR-2721, we chose a 200mg/kg dose of $^{35}S$-labelled drug so that all groups could be compared. In none of these studies could we detect a changed distribution of WR-2721 30 min after injection due to prior *cis*-Pt treatments. We analysed serum, kidney, liver, 3M2N tumour, brain and lung concentrations of WR-2721. While the amount of WR-2721 absorbed by an organ is not rigorously predictive of the level of protection which will be observed (Yuhas, 1980*b*) these studies do indicate that *cis*-Pt therapy does not alter the absorptive patterns of WR-2721, which is consistent with our observation on the lack of tumour protection.

*Multiple dose CY studies*

In an earlier report (Yuhas, 1980*b*) we demonstrated that injection of 200 mg/kg WR-2721 30 min before graded doses of CY increased the resistance of BALB/c mice to haemopoietic death by a factor of 1·5, but did not alter the CY-induced growth delays of their tumours. It is more difficult to obtain precise estimates of protection against CY when it is given repeatedly, due to the lack of a BUN-type assay and the longer interval between induction and observation of death in animals due to haemopoietic failure. For these studies, therefore, we will not attempt a comparison of protection against single *vs* multiple CY treatments, but will concentrate on the simple questions of whether WR-2721 does protect the animals against multiple treatments with CY, and whether tumour protection can be detected after multiple CY treatments.

Control BALB/c mice were given either 125 mg/kg of CY twice a week or 200 mg/kg CY once a week until death, with each treatment being preceded or not by an injection of 200 mg/kg of WR-2721 30 min earlier. In the absence of WR-2721 pretreatment these mice died an average of $36 \pm 3$ and $47 \pm 4$ days after the start of therapy, respectively. If each treatment was preceded by a WR-2721 injection, however, these survival times were increased to $61 \pm 5$ and $73 \pm 4$ days. Therefore, WR-2721 protects mice against weekly doses of CY.

Fig. 7 is a plot of the growth of the

FIG. 7.—Growth of the MCa-11 tumour in mice given no treatment (×), twice-weekly doses of 125 mg/kg of CY (●), once-weekly doses of 200 mg/kg of CY (▲), twice weekly treatments with 200 mg/kg of WR-2721 followed 30 min later by 125 mg/kg of CY (○) or once-weekly treatments with 200 mg/kg of WR-2721 followed 30 min later by 200 mg/kg of CY (▲).

MCa-11 tumour in control mice and in mice receiving the treatments listed above for non-tumour-bearing mice. No protection of the tumours is apparent in these studies, in agreement with the results listed above for *cis*-Pt (II) (Fig. 3, 4 and 6).

### DISCUSSION

From the data presented above on 3 different tumours growing in mice and rats, it is quite clear that the ability of WR-2721 to selectively protect kidneys and haemopoietic tissue but not tumours against *cis*-Pt and CY, respectively, is not only preserved under conditions of multiple treatment (daily, semi-weekly, and weekly), but is apparently enhanced, at least for *cis*-Pt. We initially expected this since it appeared that WR-2721 "neutralized" a constant amount of *cis*-Pt, as opposed to being truly dose-modifying (Yuhas & Culo, 1980). The data presented above have shown that multiple treatments are associated with larger therapeutic gains, but whether or not a constant amount of *cis*-Pt is being neutralized remains open to question. As an example, 100 mg/kg of WR-2721 increases resistance to a single dose of *cis*-Pt by a factor of 1·3 or by 1·8 mg/kg, according to the way of viewing the data. If WR-2721 were truly dose-modifying, we would have expected the 5× daily dose required to produce nephrotoxicity to rise from 1·6 mg/kg/day to 2·1 mg/kg/day, whereas if neutralization of a constant amount of *cis*-Pt were the underlying kinetics, we would have expected this dose to rise to 3·4 mg/kg/day. The observed value of 2·8 mg/kg/day lies intermediate to these two predictions, so no conclusions can be reached. Other comparisons within the data yield similar equivocal results, and resolution of this question will require more precise data.

The observations presented above brings to 6 the number of tumours which have failed to respond to the anti-chemotherapy effectiveness of WR-2721 (see above and Yuhas, 1979, 1980*b*; Yuhas & Culo, 1980). In addition, only one of the 12 tumours tested (Yuhas, 1980*d*) has been protected (DRF = 1·2) against radiation injury. This tumour, EMT6/Sf, has yielded variable results in addition to the apparent protection mentioned above (Utley *et al.*, 1974). In spite of the fact that this tumour fails to absorb appreciable quantities of WR-2721 (Utley *et al.*, 1976) just like other tumours (Yuhas, 1980*d*) it is marginally radioprotected and shows variable results in chemotherapy. Wasserman *et al.* (1980) have independently confirmed the ability of WR-2721 to offer excellent protection of the host against *cis*-Pt and CY, yet in the same hosts bearing the EMT6/Sf tumour one finds tumour protection against *cis*-Pt treatment, but no protection against CY. We conclude, therefore, that failure of WR-2721 to protect solid tumours against radiation and/or chemotherapy is fairly general but unlikely to be universal. This conclusion is reinforced by the observation of Washburn *et al.* (1974) that at least one tumour, the Morris 7777 hepatoma, absorbed WR-2721 readily, and lack of WR-2721 absorption by tumours is clearly one of the major factors involved in lack of tumour protection by this drug (Yuhas, 1980*b*, *d*). We are presently investigating the nature of this absorptive defect (Yuhas, 1980*b*; Afzal *et al.*, 1980) and hopefully will resolve the nature of this defect and be able to predict which types of tumours, if any, would be protected by WR-2721.

Research supported by Grants No. CA-21074-04 and CA-19326-03 from the National Cancer Institute, NIH, DHEW.

### REFERENCES

AFZAL, V., AFZAL, S. M. J., PARDINI, M. C. & YUHAS, J. M. (1980) The feasibility of developing central nervous system radioprotectors for use in radiotherapy. (In press.)

ECHOLS, F. S. (1974) *Normal and Malignant Tissue Response to Fractionated Radiation Exposure and the Radioprotective Drug, WR*-2721. Doctoral Dissertation, University of Florida.

ECHOLS, F. S. & YUHAS, J. M. (1976) Chemoprotection against fractionated radiation exposures with WR-2721: Skin injury. *Radiat. Res.*, **66**, 449.

JORDAN, S. W., YUHAS, J. M., KEY, C. R., BUTLER, J. & KLIGERMAN, M. M. (1979) Comparative late effects of X-rays and negative *pi* mesons on the mouse kidney. *Am. J. Pathol.*, **97**, 315.

TSUKIYAMA, I. & OHSHIMA, T. (1979) Combined effects of radioprotective and radiosensitizing drugs on normal and malignant tissues. *Abstracts 7th Int. Cong. Radiation Research.* p. 215.

UTLEY, J. F., PHILLIPS, T. L. & KANE, L. J. (1974) Differential radioprotection of euoxic and hypoxic mouse mammary tumors by a thiophosphate compound. *Radiology*, **110**, 213.

UTLEY, J. F., MARLOWE, C. & WADDELL, (1976) Distribution of $^{35}$S-labelled WR-2721 in normal and malignant tissues of the mouse. *Radiat. Res.*, **68**, 284.

WARD, J. M. & FAUVIE, K. A. (1974) The nephrotoxic effects of cis-Dichlorodiammineplatinum(II) NSC-119875 in male F344 rats. *Toxicol. Appl. Pharmacol.*, **38**, 535.

WASHBURN, L. C., CARLTON, J. E., HAYES, R. L. & YUHAS, J. M. (1974) Distribution of WR-2721 in normal and malignant tissues of mice and rats: Dependence on tumor type, drug dose and species. *Radiat. Res.*, **59**, 475.

WASSERMAN, T. H., PHILLIPS, T. L., KANE, L. J. & ROSS, G. (1980) Protection against cytotoxic chemotherapeutic effects on bone marrow colony-forming units by the radioprotector WR-2721. *Cancer Clin. Trials.* (In press.)

YUHAS, J. M. (1979) Differential protection of normal and malignant tissues against the cytotoxic effects of mechlorethamine. *Cancer Treat. Rep.*, **63**, 971.

YUHAS, J. M. (1980*a*) On the potential application of radioprotective drugs in radiotherapy. In *Radiation–Drug Interactions in Cancer Management.* Ed. Sokol. New York: Wiley & Sons.

YUHAS, J. M. (1980*b*) Active versus passive absorption kinetics as the basis for selective protection of normal tissues by WR-2721. *Cancer Res.*, **40**, 1519.

YUHAS, J. M. (1980*c*) A more general role for WR-2721 in cancer therapy. *Br. J. Cancer*, **41**, 832.

YUHAS, J. M. (1980*d*) The role of WR-2721 in radiotherapy and/or chemotherapy. *Cancer Clin. Trials*, **3**, 211.

YUHAS, J. M. & CULO, F. (1980) Selective inhibition of the nephrotoxicity of cis-dichlorodiammine-platinum by WR-2721 without altering its anti-tumor properties. *Cancer Treat. Rep.*, **64**, 57.

YUHAS, J. M., TARLETON, A. E. & HARMAN, J. G. (1978*a*) *In vitro* analysis of multicellular tumor spheroids exposed to chemotherapeutic agents *in vitro* or *in vivo*. *Cancer Res.*, **38**, 3595.

YUHAS, J. M., TARLETON, A. E. & MOLZEN, K. B. (1978*b*) Multicellular tumor spheroid formation by breast cancer cells isolated from different sites. *Cancer Res.*, **38**, 2486.